# Single-Cell Analysis of Murine Long-Term Hematopoietic Stem Cells Reveals Distinct Patterns of Gene Expression during Fetal Migration

**DOI:** 10.1371/journal.pone.0030542

**Published:** 2012-01-20

**Authors:** Jesús Ciriza, Dominique Hall, Alison Lu, Joseph Robert De Sena, Mufadhal Al-Kuhlani, Marcos E. García-Ojeda

**Affiliations:** School of Natural Sciences, University of California Merced, Merced, California, United States of America; Oklahoma Medical Research Foundation, United States of America

## Abstract

**Background:**

Long-term hematopoietic stem cells (LT-HSCs) migrate from the fetal liver (FL) to the fetal bone marrow (FBM) during development. Various adhesion and chemotactic receptor genes have been implicated in the migration of adult LT-HSCs. However, their role in the migration of fetal LT-HSCs is not clearly understood due, in part, to the rare number of these cells in fetal tissues, which preclude classical gene expression analysis. The aim of this study is to characterize the expression of migration related genes in fetal LT-HSC across different anatomical locations during development.

**Methodology/Principal Findings:**

We isolated fetal LT-HSC from different developmental stages, as well as different anatomical locations, and performed single-cell multiplex RT-qPCR and flow cytometry analysis of eight molecules involved in adult LT-HSC migration. Our results show that the gene expression of the chemokine receptor *Cxcr4* in LT-HSC varies across developmental microenvironments and times, while the cadherin *Cdh2* (*Ncad*) and the calcium receptor *Casr* show higher gene expression and variability only in FBM at 17.5 days post coitum (dpc). The cadherin *Cdh5* (*Vecad*) maintains high expression variability only during fetal development, while the integrin subunit *Itga5 (α5)* increases its variability after 14.5 dpc. The integrin subunits *Itga4* (*α4*) and *Itgal* (*Lfa1*), as well as the selectin ligand *Selplg (Psgl1)*, did not show differences in their expression in single LT-HSCs irrespective of the developmental times or anatomical microenvironments studied.

**Conclusions/Significance:**

Our data demonstrate that the expression pattern of phenotypically identical, single LT-HSCs fluctuates as a function of developmental stage and anatomical microenvironment. This is the first exhaustive gene expression comparison of migration-related molecules in fetal tissues across developmental times, enhancing the understanding of LT-HSC migration fate decisions during development.

## Introduction

Long-term hematopoietic stem cells (LT-HSCs) are characterized by their capacity to provide lifelong reconstitution of all blood cell lineages after transplantation into lethally irradiated recipients [1]. LT-HSCs in the fetal liver, as well as in the adult bone marrow (BM), can be identified by the Lineage^−/low^Sca-1^+^c-Kit^+^CD150^+^CD48^−^CD41^−^ phenotype [2], [3], [4], [5], [6]. Hematopoietic stem cells follow a defined pattern of migration through different embryonic locations during development [7]. The yolk sack as well as the aorta, gonad and mesonephros (AGM) region serve as the sites of initial hematopoiesis by 7.5 and 10 dpc, respectively. Development continues by the colonization of the FL by 11.5 dpc, followed by the FBM by 17.5 dpc (FBM17.5, reviewed in references [7], [8]). Interestingly, stem cells capable of long-term reconstitution circulate in the fetal blood as early as 12.5 dpc [8], leading to the hypothesis that circulating LT-HSC would colonize the FBM when a suitable microenvironment is present [8]. Alternatively, circulating LT-HSC might not possess the appropriate chemotactic receptor or adhesion molecule repertoire required for BM homing and migration until 17.5 dpc.

Several chemotactic receptors and adhesion molecules have been described in mice to play important roles in HSCs migration during development and adult life. The α-chemokine receptor CXCR4, specific for stromal-derived-factor-1α (SDF-1α or CXCL12), is one of the main molecules involved in LT-HSCs migration and retention in the BM [9], [10], [11], [12]. Adult HSCs migrate continuously between the BM and blood, with SDF-1α/CXCR4 signaling as one of the main regulators of this process [13]. Cadherins are calcium-dependent, homophilic adhesion proteins that comprise another group of molecules involved in LT-HSC migration. VE-cadherin is expressed in fetal hematopoietic progenitors until they reach the FL and is no longer expressed after the HSCs have migrated to the adult BM [14]. N-cadherin is expressed in both osteoblasts and quiescent HSCs [15] but its role in HSCs migration to the BM is not clear, as it has been shown that lack of N-cadherin expression does not affect stem cell maintenance [16], [17]. Additionally, the seven transmembrane-spanning calcium-sensing receptor (CASR), which responds to extracellular calcium ion concentrations, has been described to participate in HSCs migration to the BM [18]. Adult mice deficient in *Casr* have reduced numbers of HSCs in the BM, but show no differences in HSC numbers in FL at 17.5 dpc (FL17.5) when compared with wild type controls [19].

Integrins, such as α_4_β_1_ (VLA-4) and α_5_β_1_ (VLA-5), are expressed in murine HSCs [20], [21]. Fetal HSCs deficient in the integrin subunit β_1_ do not colonize the FL or adult hematopoietic tissues. Adult integrin β_1_-null HSCs fail to engraft the BM of irradiated recipient mice, remaining in circulation [22]. Furthermore, interfering with α_4_ integrin adhesion reduces the ability of HSCs to home to the BM [20]. Integrins can also act in concert to increase HSC adhesion to BM, as evidenced by the collaboration of the α_L_β_2_ integrin (leukocyte function antigen-1, LFA-1) with VLA-4 to increase HSCs adhesion [23]. Interestingly, exposure of HSCs to the chemokine SDF-1α upregulates the expression of VLA-4 and LFA-1, which in turn helps the HSCs to engraft in the BM [24]. The selectin family of adhesion proteins also mediates interactions between endothelial cells and HSCs. The P-selectin glycoprotein ligand-1 (PSGL1) mediates HSC rolling in the BM microvasculature [25]. This ligand participates in E-selectin progenitor homing by cooperating with α_4_ integrin [26].

**Figure 1:**
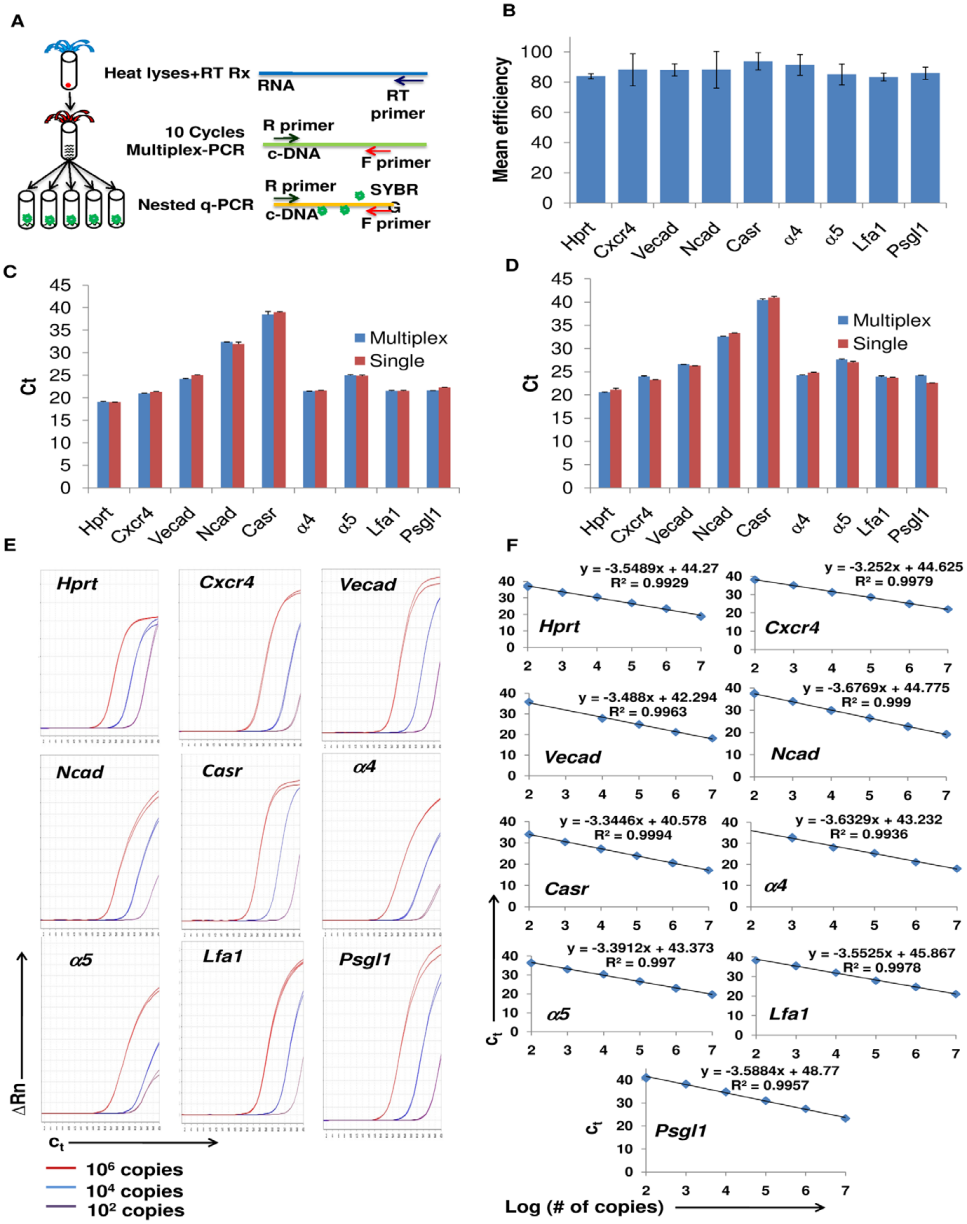
Validation of single-cell multiplex RT-qPCR. A) Schematic representation of the single cell multiplex RT-qPCR methodology. After lysing single cells by heating, cDNA is obtained by retrotranscription with as many specific primers as genes studied. The cDNA template is subjected to a 10-cycle pre-amplification reaction using a primer pair for each gene studied in order to maintain the linear ratio of the original sample. The pre-amplified product is split into aliquots for qPCR analysis with SYBR®-Green using the same primer pairs as in the pre-amplification step. B) Efficiency of qPCR reactions after pre-amplification using 10-fold dilutions of standards did not show significant differences between the genes studied. C) Similar qPCR Ct values between samples using either single or multiplex RT primers indicate no primer competition during the retrotranscription reaction. D) Similar qPCR Ct values between samples using either single or multiplex primer pairs indicate no primer competition during the 10-cycle pre-amplification reaction. E) Quantification of varied copy number for a particular gene amplified in the presence of a constant number of plasmids for the other genes studied. For each reaction, 10^2^ (purple line), 10^4^ (blue line), or 10^6^ (red line) plasmids were mixed with a constant number (10^4^) of plasmids for the other eight genes studied. Gene specific multiplex qPCR amplification was then performed. Each reaction was performed in triplicate. F) Regression lines obtained from standard curves for each gene with the single cell RT-qPCR conditions. R^2^-values were greater than 0.990 with detection limit of 100 copies for most genes.

We hypothesize that the aforementioned chemotactic receptors and adhesion molecules could be modulated throughout development during the migration of LT-HSC from the FL to the FBM. Studying the genetic mechanisms of migration presents several technical challenges that hinder classical genetic analysis. For example, our previous work showed that the number of LT-HSC in fetal tissues is highly reduced compared to the adult BM [5], precluding traditional molecular analysis. Single cell multiplex gene expression analysis provides a powerful tool to circumvent this challenge. There are two main methodologies to analyze single cell multiplex gene expression: Digital RT-PCR [27] and RT-qPCR [28]. The digital RT-PCR method requires a system such as the Fluidigm Access Array™ System and digital array chips, an uncommon and expensive technology not available to many researchers. For our study, we chose to use multiplex single cell RT-qPCR (Figure 1A) for several reasons: First, this method only requires a classical thermocycler and a qPCR system, more common and affordable instruments than the Fluidigm Access Array™ System; Second, the low number of LT-HSCs isolated from the fetal tissues yields small amount of mRNA, restricting classical gene expression studies to a few, highly expressed genes [29]; Third, population qPCR analysis reflects the average expression of a gene of interest in a population, without providing information about the distribution of gene expression by individual cells [28], [30], [31]. Single cell multiplex RT-qPCR allows for the inexpensive, simultaneous quantification of several genes of interest, illustrating the gene expression distribution by single cells within the desired population.

Our results show that the single cell pattern of expression of various genes, such as *Cxcr4*, varies across developmental and anatomical locations. Moreover, expression of *Ncad* and *Casr* is upregulated in most FBM17.5 single cells, a pattern that differs greatly from the other microenvironments studied. Furthermore, the expression of *Vecad* is downregulated after 17.5 dpc, while the expression of *α5* increases after 14.5 dpc. Taken together, our data indicate that a phenotypically identical LT-HSC population displays a dynamic gene expression pattern that varies as a function of microenvironment and developmental times.

## Results

### Multiplex RT-qPCR accurately and specifically measures differences in gene expression from single cells

Determining amplification efficiencies and discarding possible competition between pooled primers in both the retrotranscription or pre-amplification reactions are essential to validate the accuracy of the single cell multiplex RT-qPCR technique. Our results show that there were no statistically significant differences when comparing qPCR amplification efficiencies for the nine genes tested (P>0.5, Tukey test, Figure 1B). We obtained the same Ct values when single and multiplex retrotranscription reactions (Figure 1C), as well as single and multiplex pre-amplification reactions (Figure 1D), were compared. We were also able to specifically detect the fluctuation in template concentration of the genes of interest independent of the presence of other gene templates (Figure 1E). Regression curve analysis obtained R^2^ values greater than 0.99 for all studied genes, indicating high accuracy of amplification with a direct correlation between Ct value and template copy number (Figure 1F). Furthermore, we determined the limit of detection of the qPCR to be 100 copies for all genes studied, except *α4*, which had a detection limit of 1000 copies. Taken together, our data indicates that our multiplex RT-qPCR technique will allow us to accurately and confidently measure differences in gene expression from single cells.

### LT-HSCs can be detected and isolated from fetal bone marrow at day 17.5 of gestation

**Figure 2:**
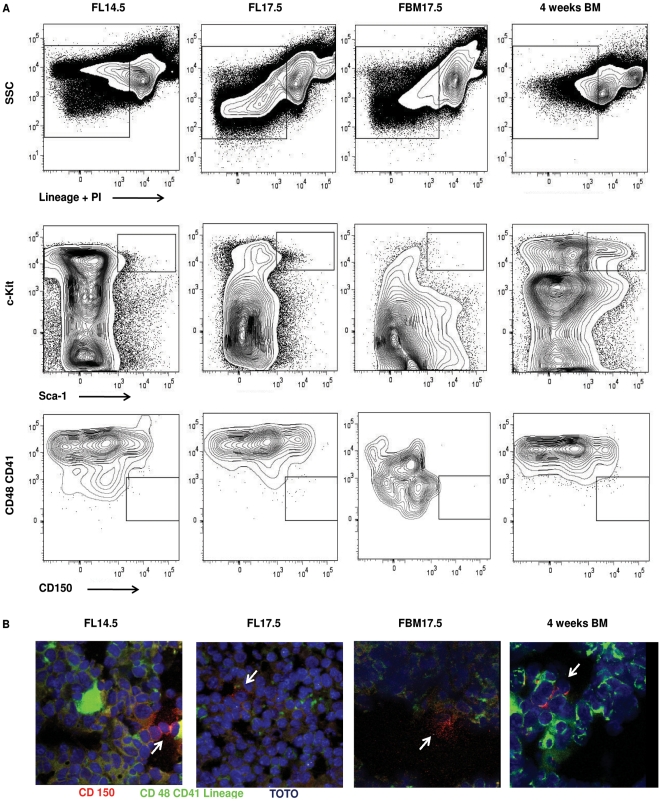
Identification of LT-HSC at different microenvironments by flow cytometry and confocal microscopy. A) Live, Lineage negative-to-low cells (gated cells in upper panels) were analyzed for the expression of c-Kit and Sca-1 (middle panels). Gates show the Lineage^−/low^ Sca-1^+^c-Kit^+^ (LSK) population, containing LT-HSC. The LSK population was then analyzed for the expression of CD150 versus CD41 and CD48 (lower panels). Gate shows LSK CD150^+^CD41^−^CD48^−^ LT-HSC. Data shown as 5% contour plots, using bi-exponential scaling and representative of three independent experiments. B) Immunohistochemistry of FL14.5, FL17.5, FBM17.5 and 4-week old adult BM. CD150 staining is shown in red while CD48, CD41 and lineage staining is shown in green. Blue staining represents nuclei marked with TOTO-3. CD150^+^CD48^−^CD41^−^Lin^−^ LT-HSCs were identified as staining only in red with blue nuclear but not green (indicated by arrow).

Previously, we identified LT-HSCs by means of flow cytometry based on the Lineage^−/low^Sca-1^+^c-Kit^+^CD150^+^CD48^−^CD41^−^ phenotype from magnetically depleted, lineage negative FL14.5, FL17.5, FBM17.5 and adult BM samples. This protocol precluded us from obtaining single LT-HSC from FBM17.5 [5]. To circumvent this problem, we eliminated the magnetic lineage-depletion step from our procedure, allowing us to isolate single LT-HSCs from fetal bone marrow. Using flow cytometry, we identified live, Lineage negative, Sca-1 and c-Kit double positive cells that constitute the Lineage^−/low^Sca-1^+^c-Kit^+^ (LSK) population (Figure 2A, upper and middle panels). We characterized LT-HSCs from the heterogeneous LSK population by gating cells with the CD150^+^CD48^−^CD41^−^ phenotype (Figure 2A, lower panels). This approach allowed us to sort an average of 5 single LT-HSCs/embryo from FBM17.5 tissue. The rest of the samples provided sufficient single LT-HSC to fill a 96-well plate/sample.

We confirmed the presence of LT-HSCs from the different tissues studied by confocal microscopy (Figure 2B and Figure S1). We identified LT-HSCs, as defined by the CD150^+^CD48^−^CD41^−^Lin^neg^ phenotype [2], in the FL14.5 and FL17.5 as well as in adult BM, detecting between 3–4 LT-HSCs/slide. However, LT-HSCs in the FBM17.5 were relatively rare, detecting about 1 LT-HSC per 5 slides. This experiment corroborated that the low number of LT-HSC in the FBM17.5 is not an artifact of flow cytometry but truly reflects the low frequency of these cells in this tissue. Overall, we were able to detect and isolate rare LT-HSCs from fetal tissues, particularly from FBM17.5 samples, for gene expression analysis.

### Single LT-HSC display varied expression levels of migration-related genes depending on developmental time and anatomical location

**Figure 3:**
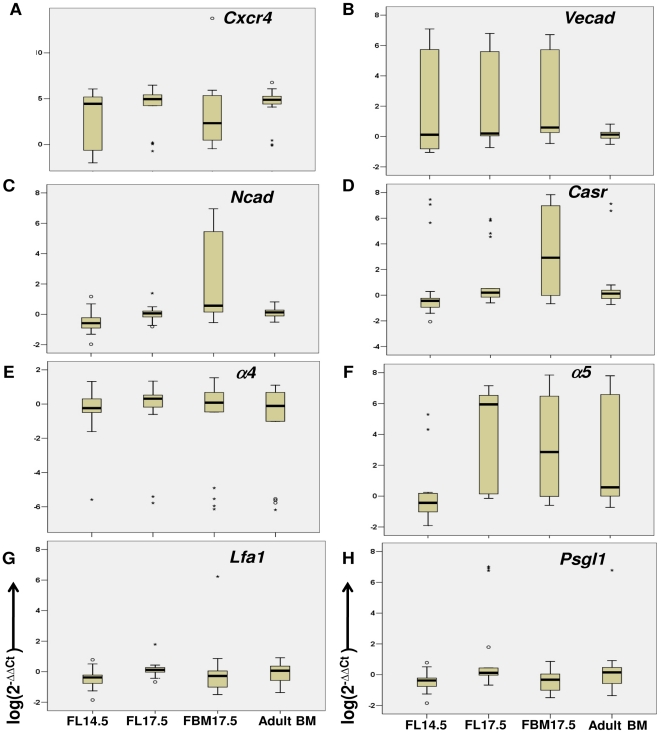
Representation of single LT-HSCs multiplex RT-qPCR analysis of genes related with their migration during development. Box plots represent the distribution of log(2^−ΔΔCt^) values of 72 single LT-HSCs from different microenvironments for each gene studied: A) *Cxcr4*; B) *Vecad*; C) *Ncad*; D) *Casr*; E) *α4*; F) *α5* G) *Lfa1* and H) *Psgl1*. Boxes represent the middle 50% of the data, localizing the 75^th^ percentile in the upper boundary of the box and the 25^th^ percentile in the lower boundary of the box. The median is shown as a bold line across the box. Vertical lines of the plot extending from the box indicate the minimum and maximum values. Circles indicate values between inner and outer fences, whereas asterisks indicate values beyond the outer fences. One single LT-HSCs from adult BM was randomly selected as reference sample.

We were interested in studying 8 genes described as essential for LT-HSCs migration: *Cxcr4, Vecad, Ncad, Casr, Itga4 (α4), Itga5 (α5), Itgal (Lfa1)* and *Psgl1*. We sorted single LT-HSCs from FL14.5, FL17.5, FBM17.5 and 4-week old adult BM to perform single cell multiplex RT-qPCR. This technique was chosen due to the low number of LT-HSCs isolated from the fetal tissues studied, particularly the FBM17.5 [5]. Whole BM RNA and sorting buffer alone were used as positive and negative controls, respectively. For each time point, embryos from three independent pregnancies, as well as BM from three independent adults, were studied (a total of nine mice per time point). Following retrotranscription and pre-amplification, we analyzed the expression of *Hprt* in each well, confirming the presence of a single cell by comparing its melting curve to the positive control (Figure S2). Samples whose *Hprt* melting curves differed from the positive control were discarded. Moreover, the expression of the gene studied was considered non-detectable if its single cell qPCR melting curve did not correspond to its respective positive control (Figure S3). The threshold value for all the genes tested was set at 2.5 to differentiate real signal from qPCR noise. The reference sample used to calculate the 2^−ΔΔCt^ values for the entire cohort of single LT-HSCs was a randomly chosen, single sorted BM LT-HSC. The data were represented by box-and-whisker plots as log(2^−ΔΔCt^), displaying the gene expression distribution of single LT-HSCs at the different time points and microenvironments examined (Figure 3).

Our data showed that the median log(2^−ΔΔCt^) expression value for *Cxcr4* among the samples was similar except for FBM17.5, which displayed a lower median value (Figure 3A). However, the distribution of *Cxcr4* expression among single cells differed, with cells from the FL17.5 and adult BM having tighter distribution and the FL14.5 and FBM17.5 displaying greater distribution. The expression of *Vecad* showed a different profile, with similar median log(2^−ΔΔCt^) values in all the samples studied but a large single cell expression distribution only in the fetal tissues studied (Figure 3B). Interestingly, many single LT-HSCs in the FBM17.5 showed higher *Ncad* and *Casr* expression than the other fetal or adult microenvironments tested (Figure 3C–D). However, while the median log(2^−ΔΔCt^) value for *Ncad* in the FBM17.5 sample was similar to the other tissues, FBM17.5 LT-HSC had a higher log(2^−ΔΔCt^) median value for *Casr*.

We also studied the expression of *α_4_*, *α_5_*, and *Lfa1* integrins, as well as the vascular addressin *Psgl1*, in all the tissues tested. The integrin subunit *α_4_*, which constitutes both the α_4_β_1_ (VLA-4) and α_4_β_7_ integrins, showed little expression differences among single LT-HSCs from the various microenvironments studied (Figure 3E). Conversely, the expression pattern of the integrin subunit *α_5_*, which constitutes the α_5_β_1_ (VLA-5), α_5_β_5_ and α_5_β_6_ integrins, was different among the samples studied. Only single LT-HSCs from FL14.5 had a tight *α_5_* expression distribution, as well as a lower median log(2^−ΔΔCt^) value. The median log(2^−ΔΔCt^) value observed in the other microenvironments varied, displaying a high expression value in the FL17.5 samples, decreasing to an intermediate expression value in the FBM17.5 sample, and further decreasing to a low expression value (comparable to the FL14.5 sample) in the adult BM LT-HSCs (Figure 3F). Lastly, no differences in *Lfa1* or *Psgl1* distribution or median log(2^−ΔΔCt^) values were detected between the microenvironments studied (Figure 3G–H). Our data show that we can successfully determine the expression levels of nine different genes from a single LT-HSC. Moreover, our analysis demonstrates gene expression variability among single cells within a phenotypically identical population, depending on developmental time and anatomical location.

### Flow cytometry analysis of migration related proteins in fetal LT-HSC

**Figure 4:**
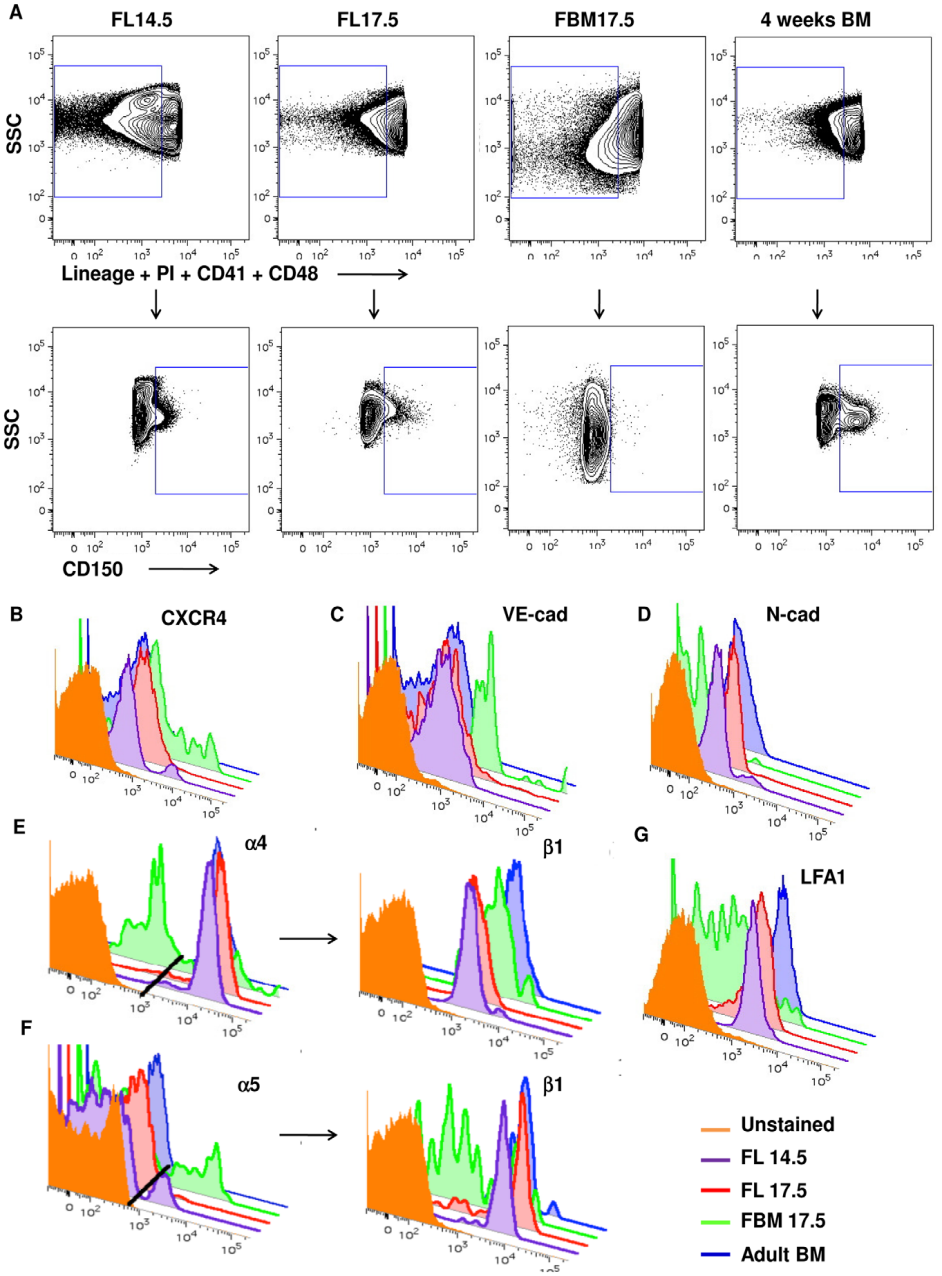
Flow cytometry analysis of proteins related with migration of LT-HSCs during development. A) Live, Lineage negative-to-low, CD41 and CD48 negative cells (gated cells in upper panels) were analyzed for the expression of CD150 (lower panels). Data shown as 5% contour plots, using bi-exponential scaling. The CD150^+^CD41^−^CD48^−^ Lineage^−/low^ LT-HSC were analyzed for the expression of B) CXCR4; C) VE-cadherin; D) N-cadherin; E) VLA-4: α4^+^ cells were gated (left histogram, gate shown as dark line) and further analyzed for expression of β1 (right histogram); F) VLA-5: α5^+^ cells were gated (left histogram, gate shown as dark line) and further analyzed for expression of β1 (right histogram) and G) LFA-1. Histograms represent expression of molecules from LT-HSCs at different microenvironments and stages of development: orange, unstained; purple, FL14.5; red, FL17.5; green, FBM17.5; and blue, 4 weeks adult BM. Data representative of three independent experiments.

We utilized flow cytometry to establish if the gene expression distributions observed by single cell multiplex RT-qPCR correlated with protein expression in the LT-HSCs populations studied. During data acquisition, only events that fell within a broad negative to medium Lineage/PI/CD41/CD48 gate and a medium to high CD150 gate were saved. For analysis of protein expression in the LT-HSC populations, we used stringent Lineage/PI/CD41/CD48^−/low^ (Lin^neg^) and CD150^high^ (CD150^hi^) gates (Figure 4A, upper and lower panels, respectively) [6]. This data acquisition strategy enabled us to obtain and analyze a statistically significant number of LT-HSCs (Lin^neg^CD150^hi^) from all of the tissues tested.

The cytometry data showed expression of CXCR4 in all the populations tested (Figure 4B), reflecting the broader distribution of *Cxcr4* expression observed by single cell multiplex RT-qPCR in LT-HSC from FL14.5 and FBM17.5 compared to FL17.5 and adult BM. VE-cadherin was expressed in most of the LT-HSCs from the fetal tissues analyzed, with the lowest expression in adult BM LT-HSCs (Figure 4C, Table S1). We found little expression of N-cadherin in the FL17.5 or adult BM LT-HSC, while a small population of FL14.5 and FBM17.5 LT-HSC expressed N-cadherin (Figure 4D). However, the mean fluorescence intensity (MFI) values for the FBM17.5 samples was almost double when compared to the MFI of the FL14.5 and other samples, indicating higher expression of N-cadherin in the FBM17.5 cells.

To examine the expression of integrins in LT-HSCs, we first gated the α_4_ or α_5_ positive populations, followed by analysis of β_1_ expression, which comprise the VLA-4 and VLA-5 populations, respectively. The vast majority of the LT-HSCs from the FL14.5, FL17.5 and adult BM were α_4_β_1_^+^, while this integrin was expressed in about 30% of the FBM17.5 LT-HSCs (Figure 4E). These α_4_β_1_^+^ populations have a tight distribution, similar to the distribution observed by multiplex single cell RT-qPCR. About 70% of β_1_^+^ cells in FBM17.5 were α_4_ negative, indicating that the FBM17.5 β_1_^+^ population includes other alpha integrin subunits. On the other hand, 7% of the α_5_β_1_ expression was confined to the FL14.5 and FBM17.5 LT-HSC populations, with few α_5_β_1_^+^ cells found in the other samples (Figure 4F). This is contrary to the multiplex RT-qPCR data where only α_5_ expression was evaluated.

Similarly to single cell RT-qPCR analysis, we did not observe differences in LFA-1 expression between microenvironments except for FBM17.5 LT-HSCs, where most of the cells were LFA-1^−/lo^ (Figure 4G). Unfortunately, we were not able to analyze the expression of CasR and PSGL1 by flow cytometry, as the antibodies available for these molecules are not suitable for this technique. Table S1 shows the mean fluorescence intensity for all the markers examined. Altogether, the flow cytometry data shows that the protein expression distribution in LT-HSC agrees with the overall single cell gene expression distribution observed by multiplex RT-qPCR among the developmental times and anatomical locations studied.

## Discussion

The molecular mechanisms that LT-HSCs use to migrate from the FL to the FBM are not well known, even when these cells follow an established pattern of migration during development [7]. The work presented here shows that we can isolate and determine the expression of eight migration-related genes from a single fetal LT-HSC by multiplex single cell RT-qPCR, accurately quantifying the single cell gene expression distribution within desired populations. We also begin to elucidate a pattern of adhesion molecules that might mediate homing of FL LT-HSC to the nascent BM vasculature as well as coordinate the migration of LT-HSC to the endosteal niche within the developing bone.

Miyamoto showed that single LSK (Lineage^−/low^Sca-1^+^c-Kit^+^) cells had a promiscuous expression of multilineage-affiliated genes [32], indicating that cells within a population with similar cell surface phenotypes might have divergent genetic expression patterns leading to different cell fates. We examined LT-HSC (LSK CD150^+^CD48^−^CD41^−^) from different anatomical locations and stages of development to quantitatively determine if a similar divergent gene expression pattern could be observed in migration-related genes. Our results demonstrate that single cells within the better-defined and more immature LT-HSC population still show a variable gene expression pattern. Nonetheless, we cannot rule out that the differences observed in our results might reflect expression differences among discrete sub-types within the LT-HSC population that inherently express these genes at different levels and not to transcriptional variability among phenotypically identical cells. Determining the single cell expression pattern of multiple housekeeping genes (for example *Actb*, *Gapdh*, and *Hprt*), believed to have more stable and uniform expression irrespective of the cells studied, could assess the latter possibility.

Studies on bone development have shown that successful migration of FL hematopoiesis to the FBM is dependent on normal embryonic bone formation [33] and endochondral ossification [34]. Initially, the primary skeleton is entirely cartilaginous [35], and the deposition of cartilage is orchestrated by the sequential maturation of chondrocytes (reviewed in [35], [36], [37]). As chondrocytes mature, they transition from a prehypertrophic state to a hypertropic state, secreting extracellular matrix that later becomes mineralized. The death of hypertrophic chondrocytes coincides with the initiation of the ossification process, which includes the vascularization of the bone, and the invasion of hematopoietic progenitors [37].

**Figure 5:**
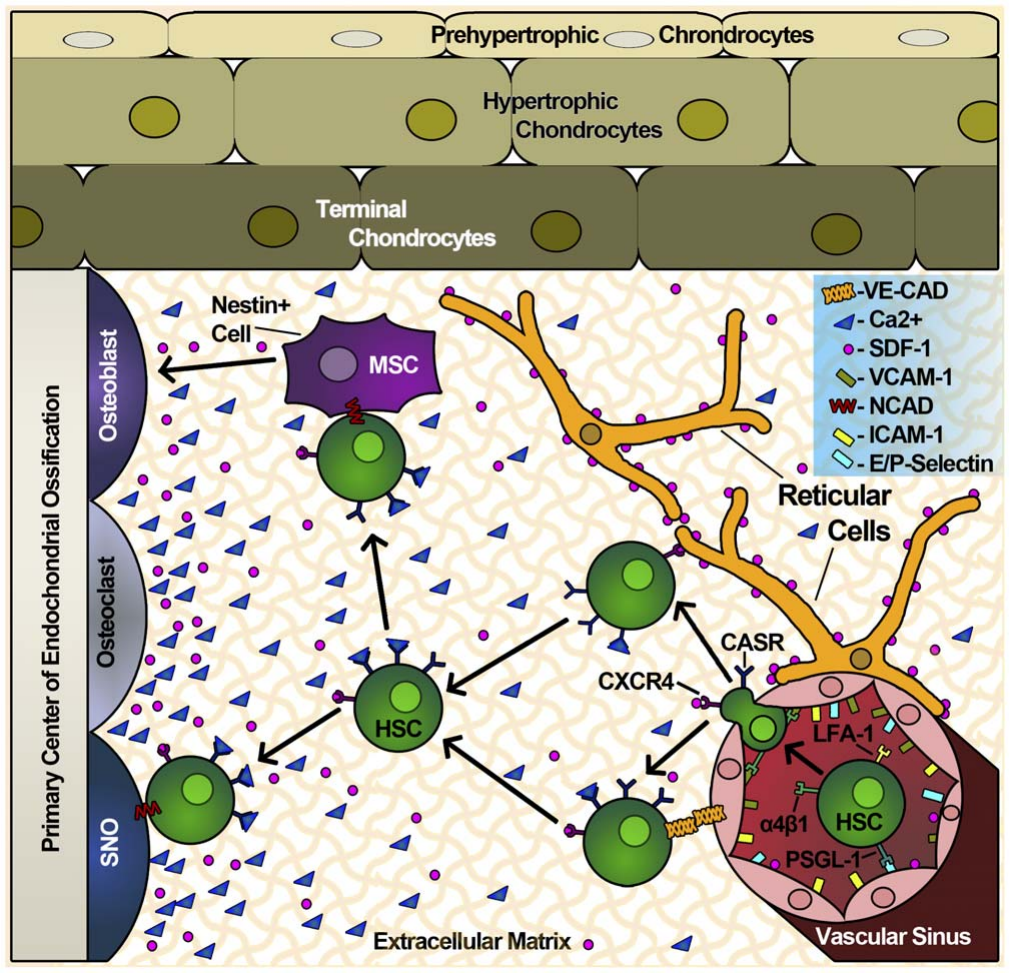
Model for the migration of LT-HSCs from the vascular niche to the endosteal niche. LT-HSC interact with bone marrow vascular endothelial cells via the PSGL-1/Selectin, allowing rolling on the vascular sinus. Stable LT-HSC adhesion occurs via VLA-4 binding VCAM-1, which also facilitates CXCR4/SDF-1α engagement and initiates endothelial transmigration. Once inside the BM, LT-HSCs interact with SDF-1α-producing reticular cells or with vascular endothelial cells. Detection of free Ca^2+^ gradients by CASR renders CXCR4 significantly more sensitive to SDF-1α, which is low in abundance [18]. Together, Ca^+2^ and SDF-1α gradients guide the LT-HSC to the endosteal niche, where they can interact with SNO (Spindle-shaped N-cadherin^+^CD45^−^ osteoblastic) cells via N-cadherin. LT-HSC migration to the endosteum might ααααααoccur via VLA-5 interaction with fibronectin in the extracellular matrix or through the action of reticular cells directing the HSC within the BM.

Our results support the idea that invasion of the fetal bone marrow by LT-HSC and other hematopoietic progenitors is a multi-step process involving chemotactic signals and multiple adhesion molecules [38] (Figure 5). Bone vascular endothelial cells constitutively express E-selectin, P-selectin, VCAM-1 and ICAM-1 in the absence of inflammation, making the BM endothelial microenvironment unique in its ability to recruit LT-HSC expressing PSGL-1, VLA-4 (α_4_β_1_) and LFA-1 (α_4_β_1_) [39], [40]. Indeed, our data shows that VLA-4 (Figure 3E and Figure 4E) and PSGL-1 (Figure 3H) are expressed in LT-HSC and that their expression does not vary significantly from anatomical location or developmental time.

Circulating LT-HSCs engage E- and P-selectins [41] on the BM vascular endothelium through PSGL-1, tethering the LT-HSC to the vascular endothelium and allowing them to roll along the vessel wall. This initial contact facilitates CXCR4-SDF-1α engagement on the extravascular space. Signals from CXCR4 are required for the migration of LT-HSC into the fetal and adult BM [10], [42], [43]. Our data shows that FL17.5 LT-HSCs uniformly express *Cxcr4* (Figure 3A and Figure 4B), allowing them to respond to SDF-1α gradients. CXCR4 signals increase the affinity of the integrins LFA-1 and VLA-4 for their ligands (ICAM-1 and VCAM-1, respectively) [24]. Engagement of VCAM-1 by VLA-4 stabilizes the interaction of the vascular endothelium and the LT-HSC, causing the latter to stop rolling [38]. Together, CXCR4 and integrin signaling induces polarization and diapedesis of LT-HSC across the BM vascular endothelium, a process mediated by LFA-1 [24]. Our RT-qPCR data show that all LT-HSC tested, regardless of their tissue of origin or developmental time, express comparable levels of *Lfa1* (Figure 3G). Surface LFA-1 expression was corroborated by flow cytometric analysis on all the populations tested. However, a lower percentage of FBM17.5 LT-HSCs were LFA-1^+^ (Figure 4G). Overall, our data suggests that PSGL-1, VLA-4, and LFA-1 are expressed at a constant level in all LT-HSC, independent of developmental time or anatomical location, and that these molecules might allow the LT-HSC to migrate into hematopoietic organs during development via vascular endothelial cell recognition.

After diapedesis, fetal LT-HSCs encounter the immature marrow microenvironment, where they interact with the basal lamina composed of extracellular matrix (ECM) proteins collagen type I, laminin and fibronectin [24]. They also interact via VLA-4 with CXCL12^+^-reticular (CAR) cells surrounding the vessel [42], or to vascular endothelial niche cells via VE-cadherin [3], [14]. Furthermore, LT-HSCs encounter both free Ca^+2^ [18], [19] and SDF-1α gradients [11], [44] that will direct them to the osteoblastic niche.

Our results show that most LT-HSC express levels of *Cxcr4* near the median of the FL14.5 sample. The large distribution and down-regulation of *Cxcr4* at this time reflects the fact that many LT-HSC are able to enter and leave the FL during this period of development. LT-HSCs with expression near the median might be retained in the FL since hematopoiesis is occurring in this organ at this developmental time. In contrast, *Cxcr4* expression in the FL17.5 shows a tight distribution near the median. At this time, the liver is transitioning from a hematopoietic to a hepatic organ [45] and many LT-HSCs are entering the circulation [8]. The tight distribution of *Cxcr4* at this time suggests that emigration of LT-HSC from the FL17.5 is mediated by a process other than down-regulation of CXCR4, such as decreased production of SDF-1α by FL stromal cells or proteolytic degradation of either SDF-1α or CXCR4 during the FL transition from a hematopoietic to a hepatic organ. In the FBM17.5, the distribution of *Cxcr4* expression resembles that of FL14.5 with a lower mean. SDF-1α plays a critical role in the attraction and retention of HSC in the BM during development [42]. CASR signaling increases the sensitivity of CXCR4 to SDF-1α in the presence of free Ca^+2^ [18], which results from the ossification of the bone at 17.5 dpc. In this way, the lower expression of *Cxcr4* that may have impaired the LT-HSC's ability to colonize the FBM17.5 environment is mitigated by increased receptor sensitivity. *Cxcr4* is upregulated in the adult BM LT-HSC where it is expressed in a tight distribution around the medium, resembling that of the LT-HSCs in the FL17.5. In the case of the adult BM, the presence of high concentrations of SDF-1α helps retain the HSCs in the bone, stabilizing hematopoiesis. Outliers in the adult BM *Cxcr4* expression might represent cells that have down-regulated CXCR4 in order to leave the BM to populate other niches (Figure 3A).

*Vecad* displays high expression variability but similar median values in all the fetal tissues tested by single cell multiplex RT-qPCR, with its expression being more uniform in adult BM LT-HSC. This finding was confirmed by flow cytometry. Our data supports and complements studies where FL Thy-1^low^Sca-1^−^Lineage^−^Mac-1^−^ cells, a population that includes LT-HSC and more mature progenitors, show decreased VE-cadherin expression by 16.5 dpc, and absence in adult BM cells [14]. We show that VE-cadherin expression is maintained in both FL and FBM samples at day 17.5 of gestation in Lineage^−/low^CD150^hi^CD48^−^CD41^−^, a more defined LT-HSC population. We propose that homotypic VE-cadherin interactions might play an important role in engaging LT-HSCs with both FL and FBM vascular endothelial niche cells during development. As the bone marrow develops into adulthood, the expression of *Vecad* diminishes (Figure 3B). This loss of VE-cadherin expression might reflect the emergence of endosteal and reticular niches as the bones are completely formed.

The role played by N-cadherin in LT-HSC interaction with its BM niches is controversial. It was previously described that N-cadherin does not play a role in adult LT-HSC adhesion to or maintenance by osteoblasts [16], [17]. Recent transplantation studies have shown that loss of N-cadherin suppresses LT-HSC engraftment [46]. However, these studies did not evaluate the role of N-cadherin during fetal LT-HSC development, migration or maintenance. Our results showed that single FBM17.5 LT-HSCs express high *Ncad* levels. This suggests that N-cadherin might be required for the initial adhesion or maintenance of LT-HSCs in the FBM, an activity that is diminished when the adult bone is completely formed. Interestingly, CAR cells express low levels of N-cadherin [9], possibly facilitating their interaction with N-cadherin^+^ FBM LT-HSC. Moreover, studies in fetal bone development show that N-cadherin is required for proper bone formation [47], and that early osteoblast development from mesenchymal stem cells can be induced through N-cadherin [48]. This raises the interesting possibility that N-cadherin expressing LT-HSC can positively influence the ontogeny of bone marrow osteoblasts during fetal development, in a manner analogous to fetal thymocytes being required for the development of the thymic medullary stroma [49], [50].

Our data suggest that initial migration and colonization of the nascent bone marrow requires the coordinated expression of the chemokine receptor CXCR4 and adhesion molecules (Figure 5). Bone marrow vascular endothelial cells express VCAM-1, E-Selectin, P-Selectin and ICAM-1 constitutively. The PSGL-1/Selectin interaction slows LT-HSC and facilitates rolling on the vascular sinus. Stable LT-HSC adhesion to endothelial cells occurs via VLA-4 binding VCAM-1, which also facilitates CXCR4/SDF-1α engagement and initiates endothelial transmigration. Once inside the BM, LT-HSCs interact with SDF-1α-producing reticular cells or with vascular endothelial cells. They are also exposed to free Ca^2+^ and SDF-1α, which are significantly less abundant in the developing BM that in the adult BM [18], [35]. Expression of CASR, the receptor for free Ca^2+^, is increased and its signals render CXCR4 significantly more sensitive to SDF-1α [18]. Together, Ca^+2^ and SDF-1α gradients guide the LT-HSC to the endosteal niche, where they can interact with SNO (Spindle-shaped N-cadherin^+^CD45^−^ osteoblastic) cells via N-cadherin. This migration might occur via the VLA-5 interaction with fibronectin in the extracellular matrix or by reticular cells directing the LT-HSC within the BM. As the BM develops and nears adulthood, *Casr* is downregulated, allowing maintenance of hematopoiesis since SDF-1α and free Ca^2+^ are abundant and BM hematopoiesis has been set into motion.

We have characterized the fetal single cell expression pattern of molecules important in migration of adult LT-HSC, and showed how this pattern fluctuates according to their developmental stage and anatomical location. Recently, embryonic stem cells have been differentiated into hematopoietic progenitors (ES-HP), which could be used in regenerative medicine of blood disorders [51], [52]. However, the therapeutic capacity of ES-HP may be limited by their ability to home and engraft to the BM. We consider that the expression pattern of migration related genes shown in this study could be useful in predicting the migration potential of hematopoietic progenitors. The present study enhances our understanding of stem cell fate decisions during migration and has potential clinical relevance that could be applied to hematopoietic stem cell or ES-HP transplantation.

## Materials and Methods

### Mice

C57BL/6 (B6) mice were purchased from the Jackson Laboratories or bred in house and housed in sterile microisolator cages with sterile feed and autoclaved water. They were euthanized by CO_2_ asphyxiation. The Institutional Animal Care and Use Committee (IACUC) at UC Merced approved all procedures.

### Tissue Isolation

Breeder mice were mated in the early evening and were checked for vaginal plugs the following morning. The morning on which vaginal plugs were observed was designated 0.5 dpc. Fetal liver and limbs were dissected from fetuses at day 14.5 and 17.5 dpc and placed in cold M199+ media (Invitrogen, Grand Island, NY) with 2% of FBS (Atlanta Biologicals, Lawrenceville, GA). Single-cell suspensions were prepared by triturating the tissues through a 70 µm nylon mesh screen. At least three pregnant females were used for each time point. Those fetuses that appeared developmentally advanced or delayed in any age group were discarded. Adult BM obtained from the hind leg bones of 4-week old mice was flushed with a 25 G needle in cold M199+ media with 2% of FBS, filtered through a 70 µm nylon cell strainer, and immediately processed for staining.

For microscopy studies, fetal liver and limbs were dissected from fetuses at day 14.5 and 17.5 dpc. Adult BM was flushed from the hind bones of 4-week-old mice. All tissues were embedded in Tissue-Tek® O.C.T. Compound (Sakura Finetek USA, Inc, Torrance, CA). Slides of five micrometers sections of OCT embedded tissues were obtained from Cureline, Inc (Burlingame, CA). Slides were store at −80°C until being processed for confocal analysis.

### Confocal Microscopy

Sample preparation and staining was performed as described by Kiel et al. [2], with some minor modifications (For details see Text S1).

### Cell staining for flow cytometric sorting and analysis

Sample preparation for cell sorting was performed as described before [5], without magnetic depletion of Lineage positive cells (For details of antibodies, fluorochromes and methodology used, see Text S1). Plates containing sorted single cells and negative samples, were stored at −20°C for 2 month as maximum or until they were used for analysis.

For flow cytometry analysis, single cell suspensions from the different tissues were stained with anti-CD16/32 antibody to block FcγII/III receptors. Fc blockage was followed by a 15 minute staining with an antibody cocktail containing PE-Cy5-conjugated anti-CD3, anti-CD4, anti-CD8, anti-CD11b, anti-CD19, anti-NK1.1, anti-Ter119, anti-GR1, anti-CD41 and anti-CD48, PE-Cy7 conjugated anti-CD150, PE-conjugated anti-CD49d, Qdot605-conjugated anti-VE-Cadherin, biotin-conjugated anti-CD49e, FITC-conjugated anti-CD11a, PE-conjugated anti-CD49d, Pacific Blue-conjugated anti-CD29, APC-conjugated anti-CD148 and Qdot525-conjugated anti-N-Cadherin. After rinsing, samples were incubated with streptavidin-conjugated APC-Alexa Fluor 750. Propidium iodide (0.5 µg/ml final concentration) was added before analysis to exclude dead cells. At least 3×10^7^ cells/sample were analyzed using a BD LSRII flow cytometer controlled by BD FACSDiva™, v.6.1.1 software (Becton Dickinson, Franklin Lakes, NJ). Unstained cells were used to evaluate autofluorescence. Each experiment was carefully compensated by single staining BD™ compBeads (BD Biosciences, San José, CA) with the appropriate antibody:fluorochrome combination, using BD FACSDiva™. In all cytometry experiments, at least three samples were analyzed for each time point.

### RNA isolation

RNA was extracted from 10^6^ adult BM cells using Trizol (Invitrogen), following manufacturer's instructions. Following DNAse I (Roche, San Francisco, CA) treatment, the concentration of RNA was determined using a Nanodrop spectrophotometer (Thermo Scientific, Waltham, MA). The RNA was used as positive control and for the setup of the conditions for qPCR analysis as well as the validation of the single cell multiplex qPCR analysis.

### Primer Design

Gene sequences for primers design were obtained from the nucleotide NCBI database [53]. Primer pairs were designed with Primer3Plus software [54], selecting oligos between 18–22 base pairs, which hybridized across introns, had similar melting temperatures, were ∼50% G/C composition, and amplified amplicons of similar size. The percent G/C of the amplified products was determined with the Bioedit software, version 7.0.5.3. Formation of primer dimers was analyzed with Primer List (http://primerdigital.com/tools/PrimerList.html). Primers specificity was tested for each primer using NCBI BLAST search engine in the mouse genome [55]. The primers selected for these PCR reactions are listed in Table 1. All primers were purchased from Sigma-Aldrich Corp, TX.

**Table 1:** Primers used for single cell qPCR analysis.

Gene name and accession number	Primer name	Primer sequence (5′→3′)
***Hprt***	Hprt RT	CAAGGGCATATCCAACAACA
NM_013556	Hprt F	GGGGGCTATAAGTTCTTTGCT
	Hprt R	GGCCTGTATCCAACACTTCG
***Cxcr4***	CXCR4 RT	GACAAAGAGGAGGTCAGCCA
NM_009911	CXCR4 F	GTGCAGCAGGTAGCAGTGAA
	CXCR4 R	GGGTTCCTTGTTGGAGTCATAG
***Vecad***	VECad RT	CGGAGGGTTGTCATTCTCAT
NM_009868	VECad F	TGGTCACCATCAACGTCCTA
	VECad R	GCACAATGGACTCTTTCCCTAC
***Ncad***	NCad RT	AGGGTCTCCACCACTGATTCT
NM_007664	NCad F	ATGATCCAAATGCCCTGAAT
	NCad R	TTTGTCCGTGACAGTTAGGTTG
***Casr***	CASR RT	ATCCTGCCTGTGATGTTACG
NM_013803	CASR F	TATCCCCCAGGTGAGCTACG
	CASR R	GATCACTTCCACCACCTGCT
***α4 integrin***	VLA4 RT	CTTGAGAGGCGATCCACAT
NM_010576	VLA4 F	CACTCCAGCCGATCCTTCA
	VLA4 R	TGCAGGCAAGCTTCACTATG
***α5 integrin***	VLA5 RT	ACCTCCTGAGGTCTCCCATC
NM_010577	VLA5 F	ATCCTGTCCGCCACTCAA
	VLA5 R	GGTCATCTAGCCCATCTCCA
***Lfa1***	LFA1 RT	CCAGCGTCATTCCCAAGTA
NM_008400	LFA1 F	AGAAGCCACCATTTCCCTCT
	LFA1 R	TGCTTGTTCGGCAGTGATAG
***Psgl1***	PSGL1 RT	GTAGGGTCAGTGGTGGCAAT
NM_009151	PSGL1 F	CTGTCACTGAGGCAGAGTCGTT
	PSGL1 R	GTTCCCGGAGATGCACAG

Note: RT primers were used for retrotranscription reaction. Forward (F) and Reverse (R) primers were used for pre-amplification and qPCR reactions. Gene accession number for each gene obtained from NCBI database (http://www.ncbi.nlm.nih.gov/nuccore) [53].

### Reverse Transcription

Sorted single cells lysed by heating at 65°C for 5 min. After cooling to 4°C, RNA from each single cell was incubated with 0.76 mM dNTPs (Invitrogen) and a cocktail containing 1.64 µM specific 3′ primers for each gene of interest for 5 minutes at 65°C. Subsequently, the RNA was retrotranscribed with 100 units of SuperScript™ III RT (Invitrogen) in a solution containing 40 units of RNase OUT™, 4.5 mM MgCl_2_, 9 mM DTT, 13 mM Tris-HCl, 32.6 mM KCl at pH 8.4 for 50 minutes at 50°C and terminated by a 5-minute incubation at 85°C. After cooling the reaction at 4°C, the samples were treated with 1 U of RNaseH for 20 minutes at 37°C and stored at 4°C. All these reactions were run on a Mastercycler® ep gradient S thermocycler (Eppendorf, Hauppauge, NY). Negative samples (without cells) and positive controls (containing 100 ng of whole BM RNA) were run alongside each retrotranscription reaction. Similar conditions were used for validating the parameters of the single cell multiplex RT analysis with whole BM RNA except for competition analysis where either single or multiplex primers were added in the retrotranscription reaction.

### Pre-amplification

All cDNAs obtained from retrotranscription reactions were diluted 4–fold in milliQ water, followed by pre-amplification with 0.05 U of Platinum® Taq DNA polymerase (Invitrogen) in 20 mM Tris-HCl (pH 8.4), 50 mM KCl, 1.5 mM MgCl_2_, 0.2 mM dNTPs and a mix containing primers pairs specific for each gene of interest, at a final concentration of 0.022 µM/primer. PCR consisted of a first step of 94°C for 45 secs and 10 cycles of amplification (1 min at 94°C, 1 min at 60°C and 1 min at 72°C). Pre-amplification reactions were run on a Mastercycler® ep gradient S thermocycler (Eppendorf). Similar conditions were used for validating the parameters of the single cell multiplex RT-PCR analysis with either whole BM RNA or cloned PCR amplicons, except for competition analysis where either primer pairs or multiplex primer pairs were added in the reaction. For details concerning cloning of PCR amplicons, see Text S1.

### Real-Time Quantitative PCR

Pre-amplified samples were diluted 5-fold before quantitative PCR studies. Real time qPCR was performed in 1× Fast SYBR® Green Master Mix (Applied Biosystems, CA) containing 5 µl of pre-amplified template and 200 nM of each specific primer pair in a StepOnePlus Real-Time PCR System (Applied Biosystems, Carlsbad, CA). After a 10 min denaturation step at 95°C, 60 cycles of amplification were performed as follows: 95°C for 3 seconds and 60°C for 30 seconds. Melting curves were generated after amplification with the following conditions: 95°C for 15 seconds, 60°C for 1 minute, a 0.3°C gradient increase and 95°C for 15 seconds. All genes, including the housekeeping gene *Hprt*, were analyzed in triplicates for each sample as well as the positive and negative controls. The same threshold value, falling on the exponential phase of the qPCR curve, was applied to all the samples amplified in order to determine the threshold cycle (C_t_). A typical experiment consisted of analyzing twenty-four cells, which displayed the correct melting curve for *Hprt*, for each tissue sample studied. Each experiment was repeated in 3 independent pregnancies per developmental time studied or 3 adult mice. The amplification efficiency, specificity and limit-of-detection qPCR experiments were performed in triplicate samples using cloned PCR amplicons as template.

### Data analysis

Flow cytometry data was analyzed using FlowJo software, version 7.2.4 or version 8.8.7 (TreeStar, Ashland, OR). Single cell quantification based in the ΔΔC_t_ method and qPCR efficiency calculations were performed using StepOne™ software, version 2.0. Confocal microscopy analysis was performed with EZ-C1 FreeViewer software, version 3.20. Tukey and Student *t*-test statistical analysis were performed using SPSS software, version 13.0.

## Supporting Information

Figure S1 **LT-HSC identification by confocal microscopy.** The immunohistochemical images of FL14.5 (1^st^ row), FL17.5 (2^nd^ row), FBM17.5 (3^rd^ row) and 4-week old adult BM (4^th^ row) are shown. Staining by CD48, CD41 and lineage markers is shown in green (1^st^ column), CD150 in red (2^nd^ column) and nuclei marked by TOTO-3 in blue (3^rd^ column). Merged images are shown in the 4^th^ column. LT-HSCs were identified as CD150^+^CD48^−^CD41^−^Lin^−^ cells.
(TIF)

Figure S2 **Verification of single cell RT-qPCR product by comparison of melting temperature curves for** ***Hprt*** **.** Single cells were sorted into 8-well strips and subjected to multiplex RT-qPCR. The melting temperature curves for the *Hprt* product were used to confirm the presence of a single cell per well and to evaluate the integrity of the sample's mRNA. Blue lines show the melting temperature curve of wells containing the positive whole BM control (300 ng RNA/sample), red lines the sorted, single LT-HSC and yellow lines the sorting buffer alone control. Wells in which the melting curve of the *Hprt* product did not match the whole BM control were discarded.
(TIF)

Figure S3 **Verification of single cell RT-qPCR product by comparison of melting temperature curves.** The melting temperature curves for each single cell RT-qPCR product (red lines) were compared to the melting temperature curve of the positive whole BM control (blue lines, 300 ng RNA/sample).
(TIF)

Table S1 **Mean Fluorescence Intensity (MFI) values.**
(DOC)

Text S1 **Supplemental Materials and Methods.**
(DOC)
