# Supplementary figures and images for: Single-Cell Analysis of Murine Long-Term Hematopoietic Stem Cells Reveals Distinct Patterns of Gene Expression during Fetal Migration
Source: PLoS One. 2012 Jan 20;7(1):e30542. doi: 10.1371/journal.pone.0030542 (PMC3262840; doi:10.1371/journal.pone.0030542)

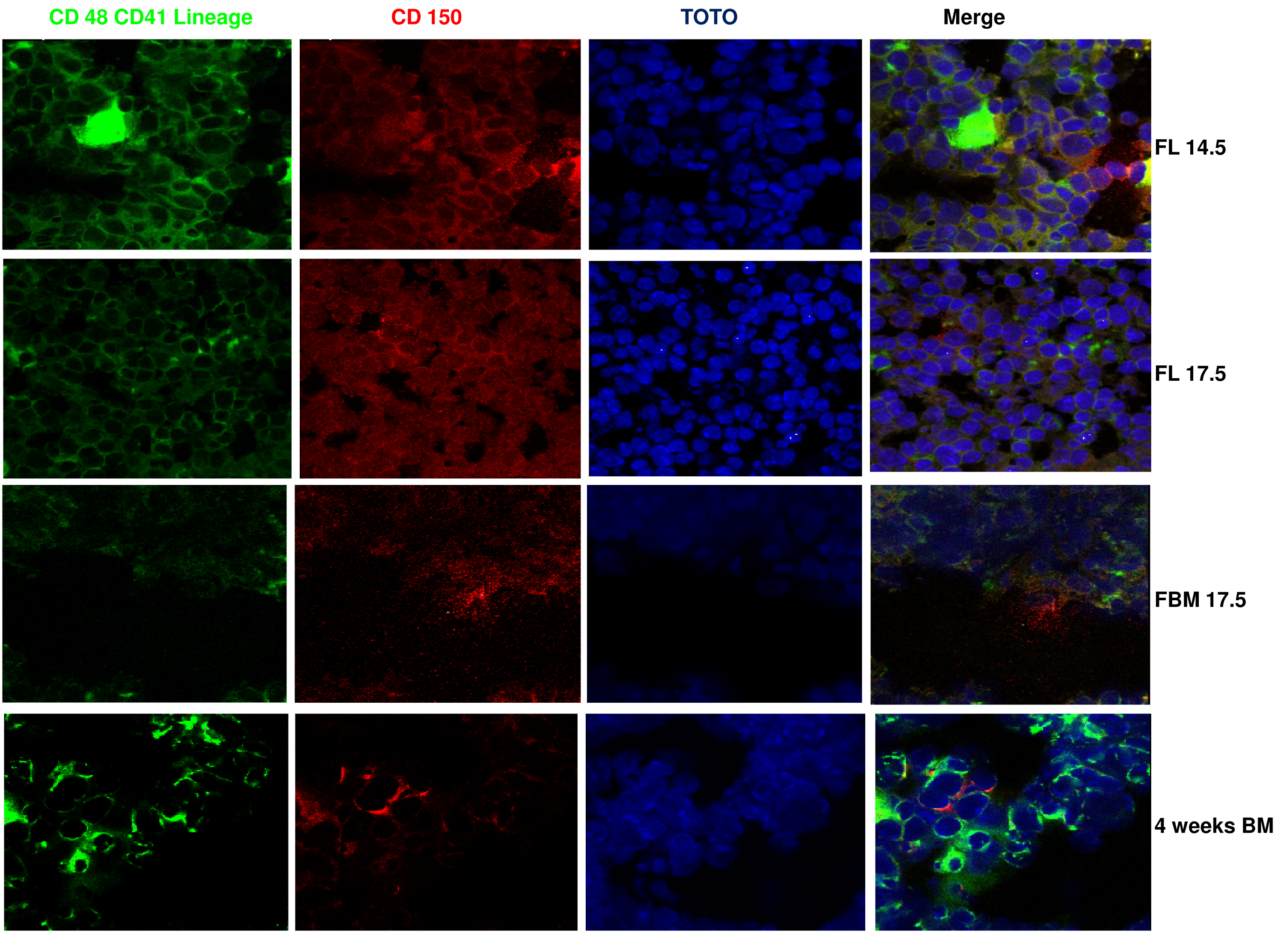

Supplement: Figure S1 — LT-HSC identification by confocal microscopy. The immunohistochemical images of FL14.5 (1st row), FL17.5 (2nd row), FBM17.5 (3rd row) and 4-week old adult BM (4th row) are shown. Staining by CD48, CD41 and lineage markers is shown in green (1st column), CD150 in red (2nd column) and nuclei marked by TOTO-3 in blue (3rd column). Merged images are shown in the 4th column. LT-HSCs were identified as CD150+CD48−CD41−Lin− cells. (TIF) [file pone.0030542.s001.tif]

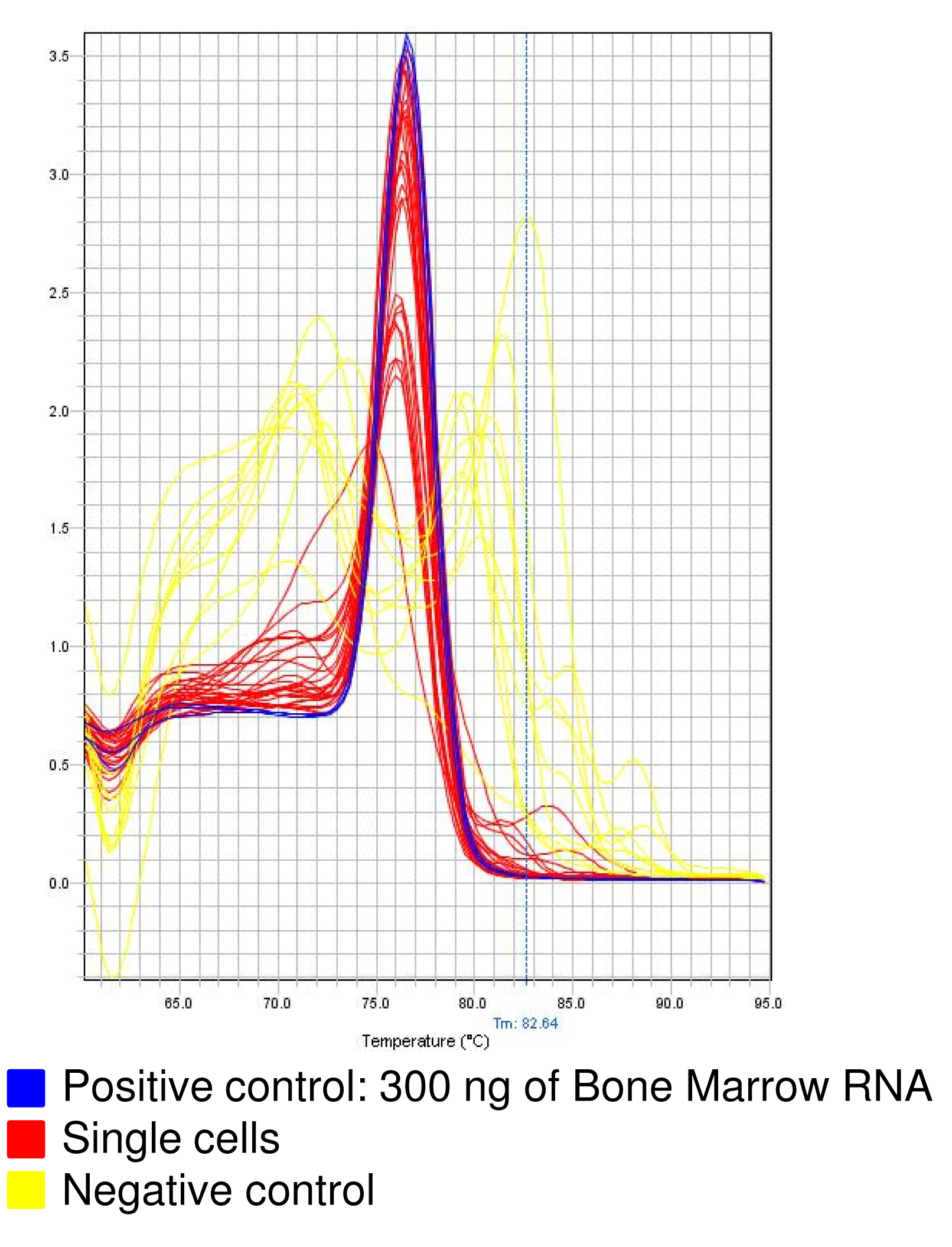

Supplement: Figure S2 — Verification of single cell RT-qPCR product by comparison of melting temperature curves for Hprt . Single cells were sorted into 8-well strips and subjected to multiplex RT-qPCR. The melting temperature curves for the Hprt product were used to confirm the presence of a single cell per well and to evaluate the integrity of the sample's mRNA. Blue lines show the melting temperature curve of wells containing the positive whole BM control (300 ng RNA/sample), red lines the sorted, single LT-HSC and yellow lines the sorting buffer alone control. Wells in which the melting curve of the Hprt product did not match the whole BM control were discarded. (TIF) [file pone.0030542.s002.tif]

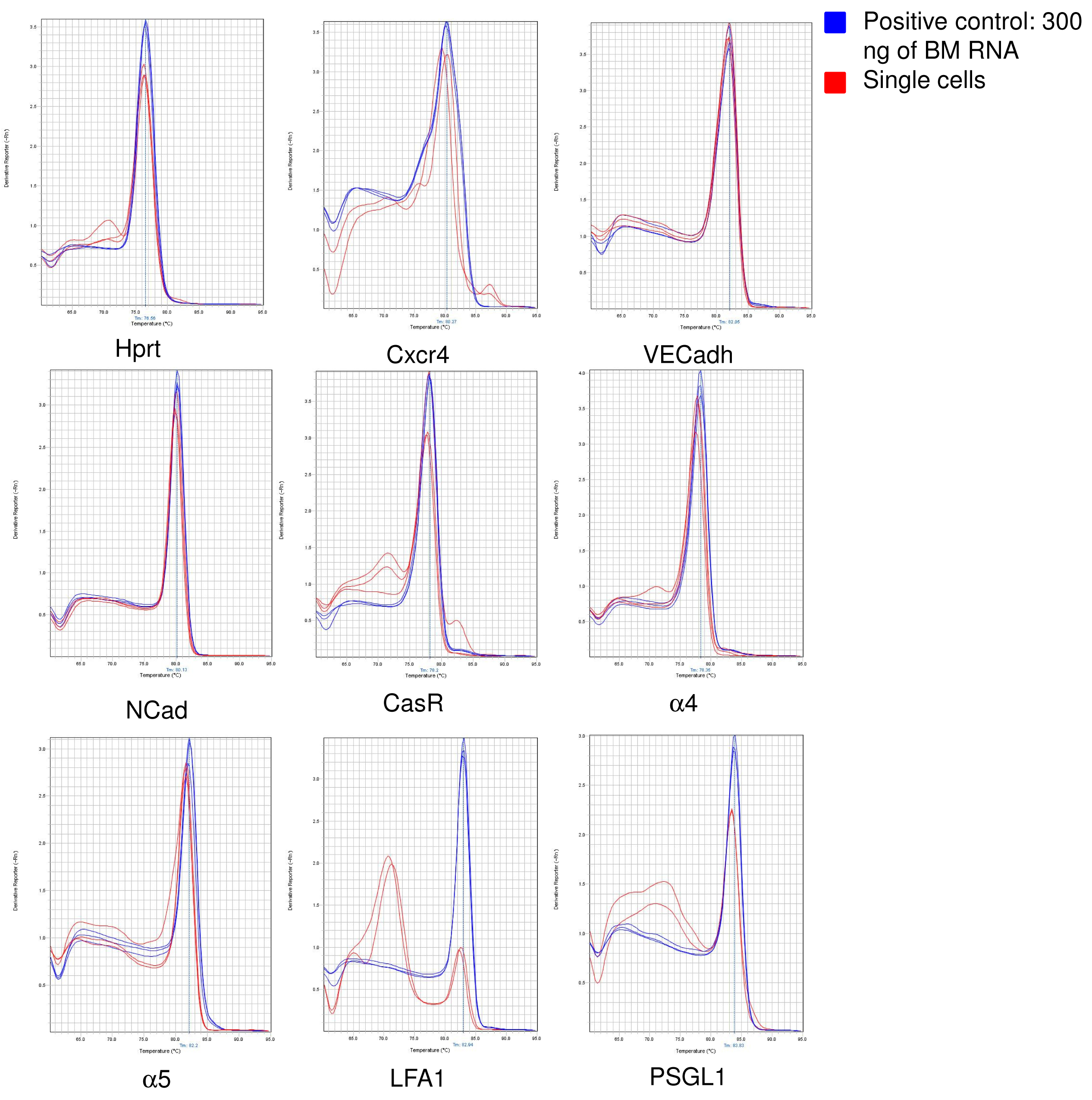

Supplement: Figure S3 — Verification of single cell RT-qPCR product by comparison of melting temperature curves. The melting temperature curves for each single cell RT-qPCR product (red lines) were compared to the melting temperature curve of the positive whole BM control (blue lines, 300 ng RNA/sample). (TIF) [file pone.0030542.s003.tif]
