# Supplementary material for: Single-Cell Analysis of Murine Long-Term Hematopoietic Stem Cells Reveals Distinct Patterns of Gene Expression during Fetal Migration
Source: PLoS One. 2012 Jan 20;7(1):e30542. doi: 10.1371/journal.pone.0030542 (PMC3262840; doi:10.1371/journal.pone.0030542)
Supplement: Table S1 — Mean Fluorescence Intensity (MFI) values. (DOC) [file pone.0030542.s004.doc]

**Table S1. Mean Fluorescence Intensity (MFI) values.**

| **Sample** | **CXCR4** | **VE-Cadh** | **N-Cadh** | **α4** | **CD29 (α4^+^)** | **LFA-1** | **α5** | **CD29 (α5^+^)** |
| --- | --- | --- | --- | --- | --- | --- | --- | --- |
| Unstained | 50.4 | 43.4 | 23.5 | 27.1 | 21.5 | 78.9 | 43.3 | 21.5 |
| FL14.5 | 447 | 394 | 462 | 17434 | 1483 | 1771 | 65.3 | 5619 |
| FL17.5 | 695 | 165 | 487 | 19330 | 3378 | 1603 | 237 | 8696 |
| FBM17.5 | 952 | 2062 | 827 | 670 | 2149 | 168 | 56 | 3931 |
| Adult BM | 251 | 82.1 | 469 | 7268 | 1260 | 1859 | 71.9 | 3931 |

Note: MFI values calculated from the samples shown in Figure 4.
