# Supplementary material for: Single-Cell Analysis of Murine Long-Term Hematopoietic Stem Cells Reveals Distinct Patterns of Gene Expression during Fetal Migration
Source: PLoS One. 2012 Jan 20;7(1):e30542. doi: 10.1371/journal.pone.0030542 (PMC3262840; doi:10.1371/journal.pone.0030542)
Supplement: Text S1 — Supplemental Materials and Methods. (DOC) [file pone.0030542.s005.doc]

**Supplemental Materials and Methods**

*Antibodies*. The following monoclonal antibodies were purchased from BioLegend (San Diego, CA) and used in flow cytometry: PE-Cy5-conjugated anti-CD3 (145-2C11), anti-CD4 (RM4-5), anti-CD8 (53-6.7), anti-CD11b (M1/70), anti-CD19 (6D5), anti-NK1.1 (PK136), anti-Ter119 (Ter119), anti-GR1 (RB6-8C5) and anti-CD48 (HM48-1), PE-conjugated anti-Sca-1 (E13-161.7) and anti-CD49d (R1-2), APC-conjugated anti-c-Kit (2B8), Alexa Fluor 647-conjugated anti-CD148 (TG12/CXCR4), PE-Cy7-conjugated anti-CD150 (TC15-12F12.2), biotin-conjugated anti-CD49e (5H10-27(MFR5)), Pacific Blue conjugated anti-CD29 (HMß1-1), FITC-conjugated anti-CD48 (HM48-1) and anti-CD11a (M17/4). Purified and FITC-conjugated anti-CD41 (MWReg30), purified anti-CD144 (eBioBV13 (BV13)), and purified anti-CD16/32 (2.4G2) were purchased from eBiosciences (San Diego, CA) and also used in flow cytometry studies. Polyclonal anti-N-Cadherin was purchased from Abcam (Cambridge, MA) to be used in flow cytometry studies. Biotin-conjugated antibodies were stained with streptavidin-conjugated APC-Alexa Fluor 750 (Caltag Laboratories, Burlingame, CA). Anti-CD41 (MWReg30) was conjugated to PECy5 by means of the Lighting-Link^TM^ PE-Cy5 Tandem Conjugation kit (Innova Biosciences, Cambridge, UK). Anti-CD144 (eBioBV13 (BV13)) was conjugated to Qdot605 nanocrystals with [Qdot® 605 Antibody Conjugation Kit](http://products.invitrogen.com/ivgn/en/US/adirect/invitrogen?cmd=catProductDetail&productID=Q22001MP&ICID=Search-Product) (Invitrogen, Grand Island, NY). Anti-N-Cadherin was conjugated to Qdot 525 nanocrystals with [Qdot® 525 Antibody Conjugation Kit](http://products.invitrogen.com/ivgn/en/US/adirect/invitrogen?cmd=catProductDetail&productID=Q22001MP&ICID=Search-Product) (Invitrogen). All the conjugations were performed following manufacture’s recommendations. Each antibody was carefully titrated and used at a concentration that gave the highest signal with the lowest background, following staining of control BM cells.

The following antibodies were used in confocal microscopy analysis: Purified anti-CD150 (TC15-12F12.2), FITC-conjugated anti-CD48 (HM48-1), anti-CD2 (RM2-5), anti-CD4 (GK 1.5), anti-CD8a (53-67), anti-CD11b (M17/4), anti-B220 (RA3-6B2), anti-Ter119 (TER-119), anti-Gr1 (RB6-8C5) were purchased from Biolegend. FITC-conjugated anti-CD41 (MWReg30) was purchased from eBiosciences. Rhodamine (TRITC)-conjugated AffiniPure Donkey Anti-Rat IgG (H+L) was purchased from Jackson ImmunoResearch (West Grove, PA) and used as secondary antibody. For nuclear staining, TOTO®-3 iodide was purchased from Invitrogen. Each antibody was carefully titrated and used at a concentration that gave the highest signal with the lowest background.

*Confocal Microscopy.* Five μm sections of FL14.5 and FL17.5, FBM17.5 and 4 week old adult BM were air-dried for 15 minutes at room temperature and subsequently fixed at -20°C acetone for 15 minutes. After rinsing, slides were blocked with blocking buffer containing 20% goat serum and 0.2% Triton-X 100 (Sigma-Aldrich, St. Louis, MO) in Dulbecco’s phosphate buffered saline (DPBS) without calcium and magnesium (HyClone, Walthan, MA) for 30 minutes prior to antibody staining. Subsequently, samples were incubated with 0.03 mg/ml purified anti-CD150 antibody in blocking buffer for 1 hour at room temperature. Following incubation, the samples were rinsed and stained for 1 hour with Rhodamine-conjugated Donkey anti-Rat reagent at room temperature to visualize the anti-CD150 antibody. After rinsing, slides were incubated with a solution of 0.1 µg/µl of purified rat IgG in blocking buffer without Triton-X 100 for 20 min at room temperature. Next, the sample was incubated for 1 hour at room temperature with a FITC-conjugated antibody cocktail prepared in the same IgG solution described above. The cocktail contained anti-CD48, anti-CD41, anti-CD2, anti-CD4, anti-CD8a, anti-CD11b, anti-B220, anti-Ter119 and anti-Gr1. Samples were then rinsed and the nuclei stained with TOTO®-3 for 20 minutes at room temperature. Finally, slides were rinsed and mounted without drying using Vectashield® mounting medium for fluorescence (Vector Laboratories, Inc. Burlingame, CA). Controls for anti-CD150 staining accuracy were done with slides stained as described above without this antibody, as well as controls without any staining. All rinse steps were done with DPBS in triplicate for 5 minutes at room temperature. Microscopy was performed using an inverted Nikon eclipse TE2000-U microscope adapted for confocal microscopy with a Nikon D-Eclipse C1 system (Nikon, Burlingame, CA). All samples were analyzed with a 30 μm aperture pinhole and 60x NA:1.40 WD:0.13 oil objective. Fluorescence was detected with an IMA 10x Argon Ion laser system (488 nm), a Green HeNe POL complete 543nm laser and a Melles Griot Red HeNe POL 633nm laser with the following filters: 515/30, 590/50 and 650LP. Images were acquired with the EC-C1 software, version 3.50. At least ten slides from three different samples were studied for each tissue.

*Cell staining for flow cytometric sorting*. In order to sort single cells by means of flow cytometry, single cell suspensions from the different tissues were stained with anti-CD16/32 antibody to block FcγII/III receptors. Fc blockage was followed by staining with an antibody cocktail containing PE-Cy5-conjugated anti-CD3, anti-CD4, anti-CD8, anti-CD11b, anti-CD19, anti-NK1.1, anti-Ter119 and anti-GR1 (Lineage markers), PE-conjugated anti-Sca-1, APC-conjugated anti-c-Kit, PE-Cy7 conjugated anti-CD150, as well as FITC-conjugated anti-CD41 and anti-CD48. Propidium iodide (0.5 μg/ml final concentration) was added before sorting to exclude dead cells. Single LT-HSCs were sorted into 96 well plate containing 20 units of RNase OUT in 10 mM Tris-HCl, 25 mM KCl at pH 8.4 (Invitrogen) using a BD FACS Aria™ Ilu flow cytometer controlled by BD FACSDiva™, v.6.1.3 software (Becton Dickinson, Franklin Lakes, NJ). Unstained cells were used to evaluate autofluorescence. Each experiment was carefully compensated by single staining BD™ compBeads (BD Biosciences, San José, CA) with the appropriate antibody:fluorochrome combination, using BD FACSDiva™. Plates containing single cells and negative samples, were stored at -20^o^C for 2 month as maximum or until they were used for analysis.

*Cloning of PCR Amplicons.* cDNA was produced from whole adult BM RNA using 5 μM oligo dT primers (Invitrogen) using SuperScript™ III First-Strand Synthesis System for RT-PCR following manufacturer’s instructions. The PCR amplification with 5 U of Platinum® Taq DNA polymerase (Invitrogen) in 20 mM Tris-HCl (pH 8.4), 50 mM KCl, 1.5 mM MgCl_2_, 0.2 mM dNTPs and the respective primer pairs for each gene (Table 1, F and R primers), at a concentration of 0.2 μM/primer. PCR consisted of a first step of 94°C for 1 min followed by 35 cycles of amplification (1 min at 94°C, 1 min at 60°C and 1 min at 72°C) and finally 10 min at 72°C. Amplification reactions were run on a Mastercycler® ep gradient S thermocycler (Eppendorf). Amplified fragments were resolved on a 2% agarose gel containing 0.5 µg/ml ethidium bromide and purified using the QIAquick® Gel Extraction Kit following manufacturer’s recommendations (Qiagen, Valencia, CA). Gel extracted DNA fragments were cloned using the pGEM®–T Easy Vector system as described by manufacturer (Promega, Madison, WI). Bacterial clones were grown overnight and plasmids were extracted using QIAprep® Spin Miniprep Kit (Qiagen). Presence of the appropriate DNA fragment was confirmed by EcoRI restriction enzyme digestion (Promega). Plasmids were quantified using a Nanodrop ND-1000 Spectrophotometer (NanoDrop Technologies, Inc, Wilmington, DE). Ten-fold serial dilutions, spanning 10^7^-10^0^ copies, were prepared for each plasmid and store at -80°C.
